# An assessment of psychological distress and professional burnout in mental health professionals in Australia during the COVID-19 pandemic

**DOI:** 10.1177/10398562211038906

**Published:** 2021-12

**Authors:** K Northwood, D Siskind, S Suetani, PA McArdle

**Affiliations:** Metro South Addiction and Mental Health Services, Queensland Health, Brisbane, QLD, Australia; School of Clinical Medicine, The University of Queensland, Princess Alexandra Hospital, Brisbane, QLD, Australia; Metro South Addiction and Mental Health Services, Brisbane, QLD, Australia; School of Clinical Medicine, The University of Queensland, Princess Alexandra Hospital, Brisbane, QLD, Australia; Physical and Mental Health Stream, Queensland Centre for Mental Health Research, Brisbane, QLD, Australia; Metro South Addiction and Mental Health Services, Brisbane, QLD, Australia; Physical and Mental Health Stream, Queensland Centre for Mental Health Research, Brisbane, QLD, Australia; Metro South Addiction and Mental Health Services, Brisbane, QLD, Australia; School of Clinical Medicine, The University of Queensland, Princess Alexandra Hospital, Brisbane, QLD, Australia

**Keywords:** mental healthcare worker, COVID-19, depression, anxiety, burnout

## Abstract

**Objective:**

To examine psychological distress and professional burnout in a cohort of Australian mental healthcare workers during the COVID-19 pandemic

**Methods:**

This study examined a multi-disciplinary cohort of mental healthcare workers in a large metropolitan service in Australia. Demographic information as well as information on employment and individual’s personal experience of the COVID-19 pandemic was collected and correlated with cross-sectional assessments of anxiety, depression and professional burnout using validated clinical questionnaires

**Results:**

Mental healthcare workers reported high levels of anxiety, depression, and professional burnout. Participants reported some reduction in anxiety since the early phases of the pandemic, but the reduction was more modest in mental healthcare workers identifying as being “vulnerable” employees.

**Conclusion:**

Despite the low numbers of COVID-19 cases, mental healthcare workers in Australia report significant levels of psychological distress and professional burnout during the pandemic.

Healthcare professionals are at higher risk of developing anxiety, depression than the general population.^1,2^ Risk factors include a challenging work environment, long working hours, high intensity work, home-work stress, and regular exposure to pain, suffering and death.^1^ Healthcare workers are also at high risk of burnout,^3^ conceptualised as a response of individuals to a stressful workplace with symptoms including emotional exhaustion, detachment, reduced fulfillment and decreased personal efficacy.^4^ Prior to the COVID-19 coronavirus pandemic, 20–80% of healthcare workers reported symptoms in keeping with professional burnout.^4^

The global COVID-19 pandemic has impacted healthcare workers as health systems internationally struggled to manage the influx of acutely unwell patients. Despite Australia and New Zealand controlling local COVID-19 transmission better than many other nations, by the end of March 2021 there had been over 30,000 COVID-19 cases and almost 1000 deaths across the two nations. Part of the management strategy has included targeted lockdowns of cities and regions, including a 112-day lockdown of Melbourne in mid-2020, and short (3–8 day) lockdowns in Perth, Auckland and Brisbane in early 2021.

Healthcare workers are on the front line of this crisis and are at higher risk of contracting COVID-19 and dying of the disease than other occupational groups.^5^ Studies have shown that during the pandemic, healthcare workers have suffered from high rates of mental distress including depression, anxiety, burnout and Post-traumatic stress disorder (PTSD).^6^ Despite the relatively lower rates of COVID-19 infection in Australia, healthcare workers have suffered from high rates of psychological distress and burnout.^7^

Mental healthcare workers have been subject to similar stressors that have affected professionals in other disciplines, including concerns about supplies of personal protective equipment (PPE) and passing infection to their families and their patients. The risk of occupational exposure to the virus may be complicated by a patient group who may struggle at times with use of PPE.

The incidence of psychological distress in the general population has significantly increased during the pandemic due to the direct impact of the virus and the socioeconomic consequences of lockdown measures.^8^ There is concern that mental health services may experience increased demand for support long after the initial phase of the pandemic has passed, and short-term investment in the mental health support sector may not completely off-set this. To date, there have been limited explorations of the risk of psychological distress and professional burnout in mental health professionals.

The aim of this study was to perform a cross-sectional assessment of levels of psychological distress and burnout in a cohort of Australian mental health professionals during the pandemic

## Methods

Metro South Human Research Ethics Committee granted ethical approval (HREC/2020/QMS/68529). Participants were recruited from mental healthcare workers at a large Queensland metropolitan mental health service. Responses were sought from medical, nursing, allied health and administrative staff. Data collection was performed using an online electronic survey, which was open for a period of two weeks in January 2021, following a “snap” lockdown in Queensland, in response to COVID-19 positive cases in the community.

Participants were invited to complete the anonymous survey on a voluntary basis via a targeted email and workplace education sessions. Data collected included demographic information, information regarding COVID-19 specific factors such as testing and isolation, Likert scales for participants to self-report the impact on the pandemic on various psychosocial aspects, and self-rated anxiety. Respondents were asked to rate their anxiety, on scale of 0–100, for two timepoints: March 2020 (retrospective, when pandemic was first declared) and January 2021 (contemporary with survey collection). Standardised and previously validated questionnaires were then used to assess levels of current (as at January 20201) anxiety, mood disturbance and professional burnout. Validated tools utilised included the Depression Anxiety Stress Scale (DASS-21), which uses 21 questions to assess symptom severity across three domains (anxiety, depression and stress)^9^, and the Copenhagen Burnout Inventory (CBI), which assesses personal, work-related and client-related burnout^10^. These tools were chosen for their ability to discriminate across dimensions of experience, their common usage in similar studies, and being relatively quick to complete, minimising burden on participants.

Data analysis was performed in the R environment. The sample population was summarised using descriptive statistics. Inferential analysis included Chi-squared analysis of proportions, ANOVA for multiple measures, and Kruskall-Wallis test statistic for ranked ordinals. Pearson correlations were performed to explore relationships between variables.

## Results

138 complete responses were included in analysis. Sample characteristics are provided in (Table 1). The respondents were predominantly female (66.1%). There was normal distribution of age. All occupational disciplines were represented, with largest group of respondents coming from a nursing background (34.8%). Medical, allied health and administrative staff with patient contact were also represented (24.6%, 26.8% and 13.8% respectively).

**Table 1.** Demographics and COVID-19 related factors of respondents

Demographics	
**Gender**	***n* (%)**
Male	46 (33.3%)
Female	91 (66.1%)
Prefer not to say	1 (0.7%)
**Age (years)**	***n* (%)**
18–24	5 (3.6%)
25–34	36 (26.1%)
35–44	35 (25.4%)
45–54	32 (23.2%)
55–64	28 (20.3%)
65+	2 (1.4%)
**Occupational discipline**	***n* (%)**
Medical	34 (24.6%)
Of which:	
- Principle House Officer (PHO)	1 (2.9%)
- Registrar	12 (35.3%)
- Senior Medical Officer/Consultant	20 (58.8%)
- Visiting Medical Officer/Locum	1 (2.9%)
Nursing	48 (34.8%)
Occupational Therapy	11 (8.1%)
Psychology	10 (7.2%)
Social Work	10 (7.2%)
Other Allied Health	6 (4.3%)
Administration	19 (13.8%)
**Covid related characteristics**	
Identify as vulnerable employee?*	***n* (%)**
Yes	33 (23.9%)
No	91 (66.6%)
Unsure	13 (9.5)
**Number of days leave for Covid testing/isolation^**	***n* (%)**
0	55 (39.9%)
1–4	67 (48.5%)
5–9	11 (7.8%)
>10	5 (3.8%)
	**Median, IQR, Max**
	1, 3, 25
**Number of Covid-19 tests completed**	***n* (%)**
0	52 (37.7%)
1–4	83 (60.1%)
5–9	3 (2.2%)
>10	0 (0%)
	**Median, IQR, Max**
	1, 2, 7
**Ever received a positive Covid-19 test?**	***n* (%)**
Yes	0 (0%)
No	138 (100%)
**Common concerns about Covid-19**	***n* (%)**
Risk of becoming infected in the workplace	98 (71%)
Risk of passing on infection onto others (such as family)	105 (76.1%)
Concern about PPE or working conditions	87 (63.0%)
Increased workload	88 (63.8%)
Increased need to cover emergent leave	80(58.1%)
Fear of redeployment to other services	24(17.4%)

* “Vulnerable employee” defined according to the Australian Government Department of Health guidelines (https://www.health.gov.au/news/health-alerts/novel-coronavirus-2019-ncov-health-alert/advice-for-people-at-risk-of-coronavirus-covid-19) as at the time of data collection, self-identified by respondents.

^Does not include days where respondents worked from home while in isolation / on sick leave.

IQR = Interquartile Range.

With respect to COVID-19-related factors, a significant proportion (23.9%) self-identified as a “vulnerable” employee according to the federal government guidelines. 59.8% of respondents reported at least one day of absence for COVID testing/isolation (median 1 day, interquartile range 3, max 25 days). 62.3% of respondents reported at least one COVID test (median 1 day, interquartile range 3, max 7). No respondent reported a positive test result. Respondents identified a number of key concerns related to working during the COVID 19 pandemic, including risk of being infected in the workplace, passing infection to others, and concerns about increased workload.

**Figure 1.**
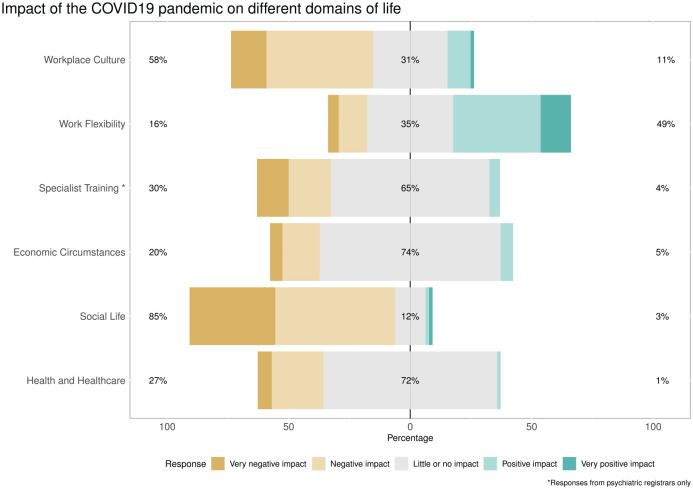
Likert scales of the impact of the COVID-19 pandemic on multiple domains of life. The majority of respondents reported that the pandemic had a negative or very negative effect on their social lives and workplace culture, while also feeling that it has positively or very positively impacted on their work flexibility. For most respondents, there was neutral impact on economic circumstances.

Likert scales were used to assess impact of the pandemic on various dimensions of life (Figure 1); The majority of respondents felt that the pandemic had had a negative impact on their workplace culture (encompassing raised workload intensity, high acuity of patients, and need to cover emergent leave), however this was contrasted with a perceived benefit of increased work flexibility afforded by telehealth/working from home. Amongst psychiatry trainees, the majority reported negative impact on training and career progression. Most found that the pandemic had negatively impacted their social life, but health/healthcare and economic impacts were mostly neutral for this cohort. Some respondents reported negative economic impact resulting from loss of a partner’s employment, adult children returning to the home and requiring support, and the non-renewal of temporary contracts. Sample comments regarding impacts are provided in (Table 2).

**Table 2.** Qualitative themes from free-text comments about the impact of COVID-19 on domains of life

**Workplace culture**
***Predominant theme*** Increased workload compounded by emergent sick leave, low morale, understaffing and lack of backfill ***Quotes*** “increased burnout and stress due to low staffing” “workload has increased as backfill is no longer possible due to budget cuts” “Inability to provide adequate clinical care due to COVID-19 restrictions” “morale was very low”
**Work flexibility**
***Predominant theme*** Those who could work from home felt that this was beneficial. Frustration from other occupational groups who were not offered the option of remote working ***Quotes*** “working from home actually increased my productivity. Video conferencing has saved much time. The team see the positive aspects of working from home and are keen to keep this as an option” “inpatient services were unable to work from home. Inpatient felt this was unfair as the risk of exposure was greater” “Being forced to break down life compartments eg install work programs on my home devices, having 24/7 team communication via social media apps”
**Specialist training**
***Predominant theme*** Difficulties accessing training opportunities and delays in sitting professional exams ***Quotes*** “Courses cancelled, unable to hold groups” “inability to progress through training” “unable to sit certain exams etc”
**Economic Circumstances**
***Predominant theme*** Little direct impact on MHCW’s but having to support others whose financial situation has deteriorated during the pandemic ***Quotes*** “adult children have both moved home due to job loss resulting in increased living expenses” “having to financially support others in the house hold directly affected with loss of employment hours” “less work for my husband due to covid” “my partners business shut down”
**Social Impact**
***Predominant theme.*** Loss of contact with friends and family. Unable to attend family events. Loss of ability to recuperate with a holiday ***Quotes*** “unable to attend a funeral, unable to check on physically failing parents, had to cancel holiday” “unable to visit older family members, unable to attend wedding interstate” “waited 50 years for our trip of a lifetime- had to be cancelled” “wedding delayed, trips cancelled”
**Health and Healthcare**
***Predominant theme*** Negative impact on mental health. Flexibility in accessing services via telehealth a benefit. Difficult to access services on a face-to-face basis ***-Quotes*** “physical health has had no impact, but mental health has had a negative impact” “telehealth was very useful for our family. Psychology appointments and GP etc” “slight negative impact with specialists appointments being via telephone”

All quotes are verbatim, including spelling and punctuation.

**Figure 2.**
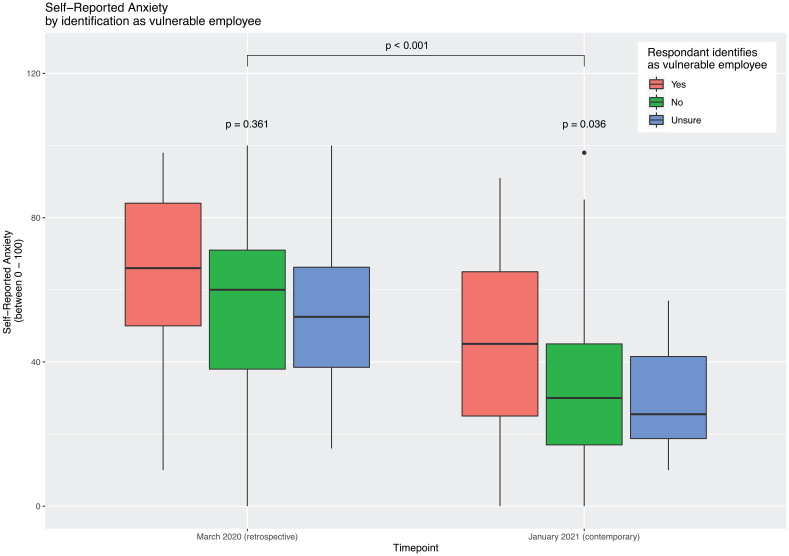
Relationship between self-reported anxiety and identification as vulnerable employee. Participant’s retrospectively self-rated their anxiety as at March 2020, and at the time of data collection in January 2021. There was a significant decline in self-reported anxiety across the two time points(*p* < 0.001), but this reduction was more modest in patients who identified as “vulnerable” compared to those who did not, or were unsure of vulnerability status (*p* = 0.036). This difference in anxiety was not present in March 2020 (*p* = 0.361).

There was a statistically significant reduction in self-reported anxiety between the March 2020 and January 2021 (*p* < 0.001, Figure 2). Amongst vulnerable-identifying employees, the reduction was less marked, and they retained significant anxiety compared to non-vulnerable employees (*p* = 0.036).

**Figure 3.**
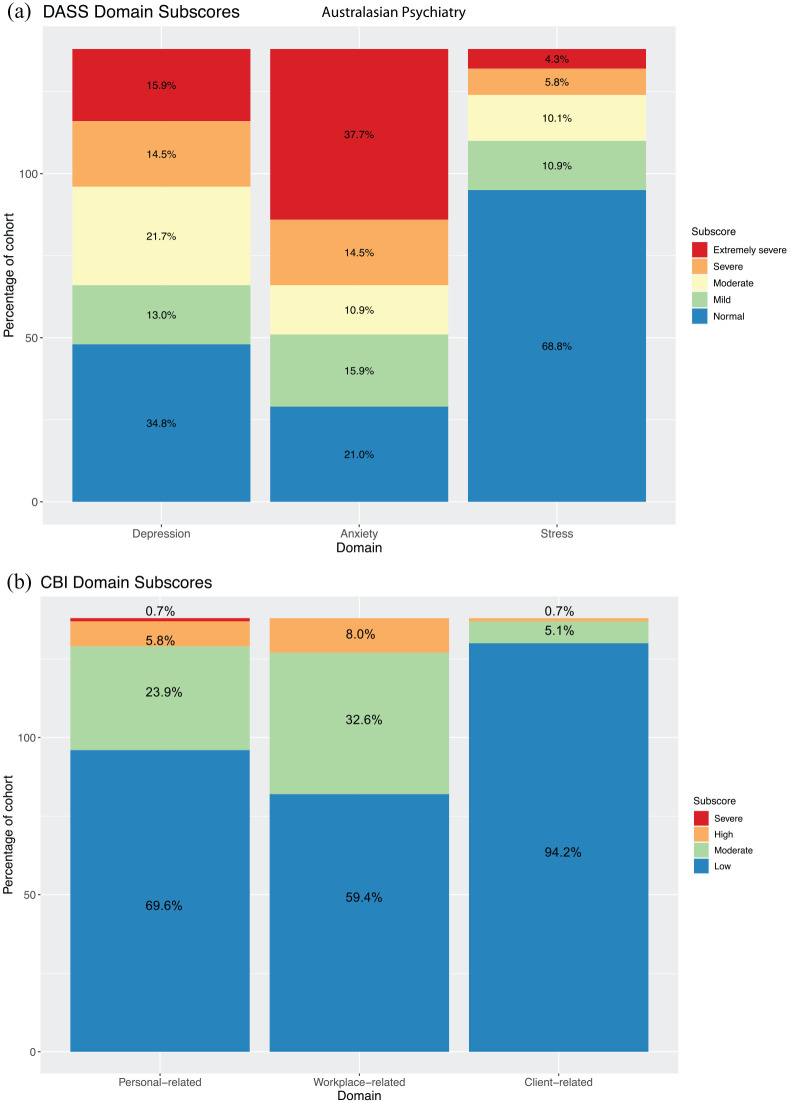
Domain subscores for validated Depression, Anxiety and Stress Scale (DASS) and Copenhagen Burnout Inventory (CBI). (a) Breakdown of DASS subscores reveal that a majority of respondents report moderate or higher depression and anxiety scores (52.1% and 63.1% respectively). Moderate or higher stress related subscores were reported in 42% and (b) CBI subscores show a moderate or higher personal and workplace-related burnout in 30.4% and 40.6% of respondents respectively. Client-related burnout scores remained low for the overwhelming majority (94.2%).

DASS and CBI scores for respondents revealed elevated depression and anxiety sub-scores within the cohort, with 52.2% and 63.0% of respondents reporting moderate or more severe levels of depression or anxiety, respectively (Figure 3). In contrast, only 20.3% had a moderate or higher stress sub-score. Respondents’ self-rated anxiety, as discussed above, correlated with their score in the anxiety subdomain for DASS for both time measures, but the strongest relationship was to the March self-measure (*R* = 0.45, *p* < 0.001). Scores suggestive of moderate or more severe burnout for personal and workplace subdomains were 30.4% and 40.6% respectively, but patient-related burnout was low, at <10%. There were no significant differences in any of the subdomains by gender, occupational discipline or any other factor for either DASS or CBI scores.

## Discussion

The findings from this study suggest that the COVID-19 pandemic has had negative impacts on mental healthcare workers across multiple life domains, particularly in relation to their workplace culture and social activities. Some mental healthcare workers perceived benefits related to improved work flexibility by increased use of telehealth and the ability to undertake work remotely from home. The themes identified in this work may be helpful for developing workplace supports in the future.

Self-reported anxiety was high at the start of the pandemic but had reduced by early 2021, possibly due to Australia’s effective pandemic management, improved understanding of the virus, and increased PPE availability. The retrospective collection of this self-reported measure may affect validity of this measure. Interestingly, although most workers reported a longitudinal reduction in anxiety, workers identifying as vulnerable reported smaller reductions compared to those who did not consider themselves to be vulnerable. It is not known if vulnerable employees were supported in line with government recommendations, which may have affected their anxiety levels. More than half of the cohort reported currently experiencing moderate to extremely severe symptoms in their DASS anxiety and depression sub-scores. CBI scores indicate high levels of personal and workplace burnout, but less patient-related burnout, suggesting that mental healthcare workers still find patient contact rewarding during the pandemic.

As the current study is cross-sectional in nature, it is unclear to what extent reported psychological distress and professional burnout is directly attributable to the pandemic. Reduction in anxiety levels since the pandemic began suggests that this may be a direct effect of the pandemic. While uncertainty still remains around the pandemic, increasing vaccine availability and ongoing effective virus containment in Australia may support future reduction in levels of distress amongst mental healthcare workers. However, medium-long term socioeconomic impacts on the patient population may result in sustained demand on mental health services, contributing to ongoing distress. Further exploration of these varied impacts will be important to support the mental health workforce.

## Conclusion

Our findings suggest that the COVID-19 pandemic has had a significant impact on the emotional wellbeing of mental healthcare professionals, resulting in anxiety and distress. We suspect that this will improve with time, mirroring the trajectory of the pandemic in Australia, but uncertainty remains. Regular monitoring and evaluation of the psychological distress among mental health professionals is warranted moving forward.
